# Supplementary material for: The Mitochondrial Distribution and Morphology Family 33 Gene FgMDM33 Is Involved in Autophagy and Pathogenesis in Fusarium graminearum
Source: J Fungi (Basel). 2024 Aug 16;10(8):579. doi: 10.3390/jof10080579 (PMC11355928; doi:10.3390/jof10080579)
Supplement: Supplementary file 1 [file jof-10-00579-s001.zip › legend.pdf]

Figure S1. Targeted gene replacement of *FgMDM33* and confirmation by Southern blotting hybridization. **A,** Construction of the DNA fragments for *FgMDM33* knockout and the targeted replacement pf *FgMDM33*. B: Bgl I. **B,** Southern blotting analysis. Top panel: genomic DNA was digested with Bgl I and probed with an *HPH* cassette (approximately 1.5 kb). Bottom panel: genomic DNA was digested with Bgl I and probed with partial *FgMDM33* replacement (approximately 1.1 kb).

Figure S2. Amino acid alignment and phylogenetic analysis of FgAif1 with its orthologs from other fungal species. **A,** Alignment of the amino acid sequence of FgAif1 with its orthologs from other fungal species. **B,** Phylogenetic tree. The analysis was performed using the program MEGALIGN. GenBank accession numbers: *Aspergillus niger* ANI_1_80104, *Botrytis fragariae* Bfra_002363, *Colletotrichum fructicola* CGMCC3_g4985, *C. siamense* CGCS363_v012823, *Fusarium culmorum* FCULG_00011295, *F. graminearum* FGSG_02433, *F. proliferatum* BFJ72_g7824, and *Rhizoctonia solani* RhiXN_06589.

Figure S3. The yeast two-hybrid assays. The negative reaction among FgMdm33 and FgMmm1, FgMdm10, FgMdm12, FgMdm34, FgPhb1, and FgPhb2, respectively. The fusion constructs were co-introduced into the yeast Y2H Gold strain. All transformants were cultured on SD/-Ade/-Leu/-Trp/-His plates for 3 days. The pair of plasmids pGADT7-T and pGBKT7-53 was used as the positive control, and the pair of plasmids pGADT7-T and pGBKT7-Lam was used as the negative control. AD, pGADT7; BD, pGBKT7.

Table S1. Primers used in this study.
