# Supplementary material for: The Mitochondrial Distribution and Morphology Family 33 Gene FgMDM33 Is Involved in Autophagy and Pathogenesis in Fusarium graminearum
Source: J Fungi (Basel). 2024 Aug 16;10(8):579. doi: 10.3390/jof10080579 (PMC11355928; doi:10.3390/jof10080579)
Supplement: Supplementary file 1 [file jof-10-00579-s001.zip › Table S1.pdf]

Table S1. Primers used in this study.

Primer	Sequence (5'-3')	Relevant characteristics
33AD-F<br>33AD-R	CATATGGCCATGGAGGCCAGTGAATTCATGCAGCCGTTCGCTCGTCC<br>CGATGCCCACCCGGGTGGAATTCTCATCTTCGAACCATTAGTA	Amplification of the full-length cDNA sequence of *FgMDM33* for fusion with pGADT7 plasmid
33BK-F<br>33BK-R	CATATGGCCATGGAGGCCGAATTCATGCAGCCGTTCGCTCGTCC<br>CGACGGATCGCCGGGAATTCTCATCTTCGAACCATTAGTA	Amplification of the full-length cDNA sequence of *FgMDM33* for fusion with pGBKT7 plasmid
1AD-F<br>1AD-R	CATATGGCCATGGAGGCCAGTGAATTCATGGCTGGTGATACTTGTCC<br>TCGATGCCCACCCGGGTGGAATTCTAACTATGGAGTCGGGATCT	Amplification of the full-length cDNA sequence of *FgMMM1* for fusion with pGADT7 plasmid
1BK-F<br>1BK-R	CATATGGCCATGGAGGCCGAATTCATGGCTGGTGATACTTGTCC<br>CGACGGATCGCCGGGAATTCTAACTATGGAGTCGGGAT	Amplification of the full-length cDNA sequence of *FgMMM1* for fusion with pGBKT7 plasmid
10AD-F<br>10AD-R	CATATGGCCATGGAGGCCAGTGAATTCATGCGCGAGTTTATGGACTA<br>TCGATGCCCACCCGGGTGGAATTCTTATGATGAAAACTGAATTT	Amplification of the full-length cDNA sequence of *FgMDM10* for fusion with pGADT7 plasmid
10BK-F<br>10BK-R	CATATGGCCATGGAGGCCGAATTCATGCGCGAGTTTATGGACTA<br>TCGACGGATCGCCGGGAATTCTTATGATGAAAACTGAAT	Amplification of the full-length cDNA sequence of *FgMDM10* for fusion with pGBKT7 plasmid
12AD-F<br>12AD-R	CATATGGCCATGGAGGCCAGTGAATTCATGTCAATTGAATTAAACTG<br>TCGATGCCCACCCGGGTGGAATTCTTACACCAAAAAGGTCCAGA	Amplification of the full-length cDNA sequence of *FgMDM12* for fusion with pGADT7 plasmid
12BK-F<br>12BK-R	CATATGGCCATGGAGGCCGAATTCATGTCAATTGAATTAAACTG<br>TCGACGGATCGCCGGGAATTCTTACACCAAAAAGGTCCAG	Amplification of the full-length cDNA sequence of *FgMDM12* for fusion with pGBKT7 plasmid
34AD-F<br>34AD-R	CATATGGCCATGGAGGCCAGTGAATTCATGGCCTTCAACTTCAACTG<br>GATGCCCACCCGGGTGGAATTCTTATCGAGTAGCTGCCTC	Amplification of the full-length cDNA sequence of *FgMDM34* for fusion with pGADT7 plasmid
34BK-F<br>34BK-R	CATATGGCCATGGAGGCCGAATTCATGGCCTTCAACTTCAACTG<br>GACGGATCGCCGGGAATTCTTATCGAGTAGCTGCCT	Amplification of the full-length cDNA sequence of *FgMDM34* for fusion with pGBKT7 plasmid
P1AD-F	CATATGGCCATGGAGGCCAGTGAATTCATGGCTGGCGCTGCACGAG	Amplification of the full-length

P1AD-R	TCGATGCCCACCCGGGTGGAATTCTTAAGCCCTGCCCACTGACA	cDNA sequence of *FgPHB1* for fusion with pGADT7 plasmid
P1BK-F<br>P1BK-R	CATATGGCCATGGAGGCCGAATTCATGGCTGGCGCTGCACGAG<br>TCGACGGATCGCCGGGAATTCTTAAGCCCTGCCCACTGACA	Amplification of the full-length cDNA sequence of *FgPHB1* for fusion with pGBKT7 plasmid
P1AD-F<br>P2AD-R	CATATGGCCATGGAGGCCAGTGAATTCATGTCGAACAACAACTGGCA<br>CGATGCCCACCCGGGTGGAATTCCTAGCTCTTATCGTTTTTGT	Amplification of the full-length cDNA sequence of *FgPHB2* for fusion with pGADT7 plasmid
P2BK-F<br>P2BK-R	CATATGGCCATGGAGGCCGAATTCATGTCGAACAACAACTGGCA<br>TCGACGGATCGCCGGGAATTCCTAGCTCTTATCGTTTTTGT	Amplification of the full-length cDNA sequence of *FgPHB2* for fusion with pGBKT7 plasmid
33-UF<br>33-UR	TCGGTCAGGTAGCGAAGA<br>TCCACTAGCTCCAGCCAAGCCAAGACGGCTAAAGG	Amplification of upstream fragment of *FgMDM33* for the gene deletion
33-DF<br>33-DR	AGTAGATGCCGACCGAACAACTGTAGGGCTGGTAGTCG<br>TGGAGGCAGTTCGTGTTA	Amplification of downstream fragment of *FgMDM33* for the gene deletion
33-NF<br>33-NR	ACATACCTGTCGTCGTGG<br>CAAAGAGGCTGAGCTACA	Amplification of the fragment, including full length ORF of *FgMDM33* and its upstream and downstream sequences
33YW-F<br>33YW-R	CGCAACATCGTGTCAGTG<br>CAAGAAGTTCATGCCCATT	For identification of *FgMDM33* gene deletion mutants (presence or not), or *FgMDM33* complemented transformants
33DX-F<br>33DX-R	TGCTATTACCTGGTTGAG<br>GGCTACCCTGGAAGATGG	For identification of *FgMDM33* gene deletion mutants (size difference)
33Com-F<br>33Com-R	GGTACCCGGGATCCTCTAGAGGTATGAGCCGACGTGAGT<br>TGCCTGCAGGTCGACTCTAGACAGTAAAGTGATGTTGCGTTGG	Amplification of fragment including full length ORF of *FgMDM33* and its native promoter and terminator DNA sequences for the complementation
