# Supplementary figures and images for: The Mitochondrial Distribution and Morphology Family 33 Gene FgMDM33 Is Involved in Autophagy and Pathogenesis in Fusarium graminearum
Source: J Fungi (Basel). 2024 Aug 16;10(8):579. doi: 10.3390/jof10080579 (PMC11355928; doi:10.3390/jof10080579)

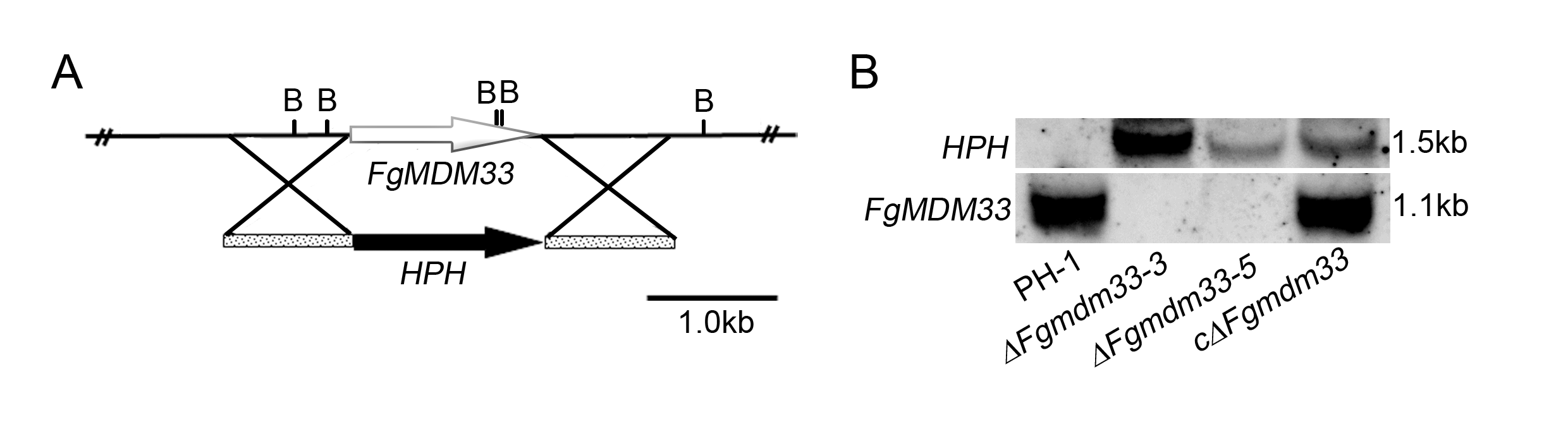

Supplement: Supplementary file 1 [file jof-10-00579-s001.zip › Figure S1.tif]

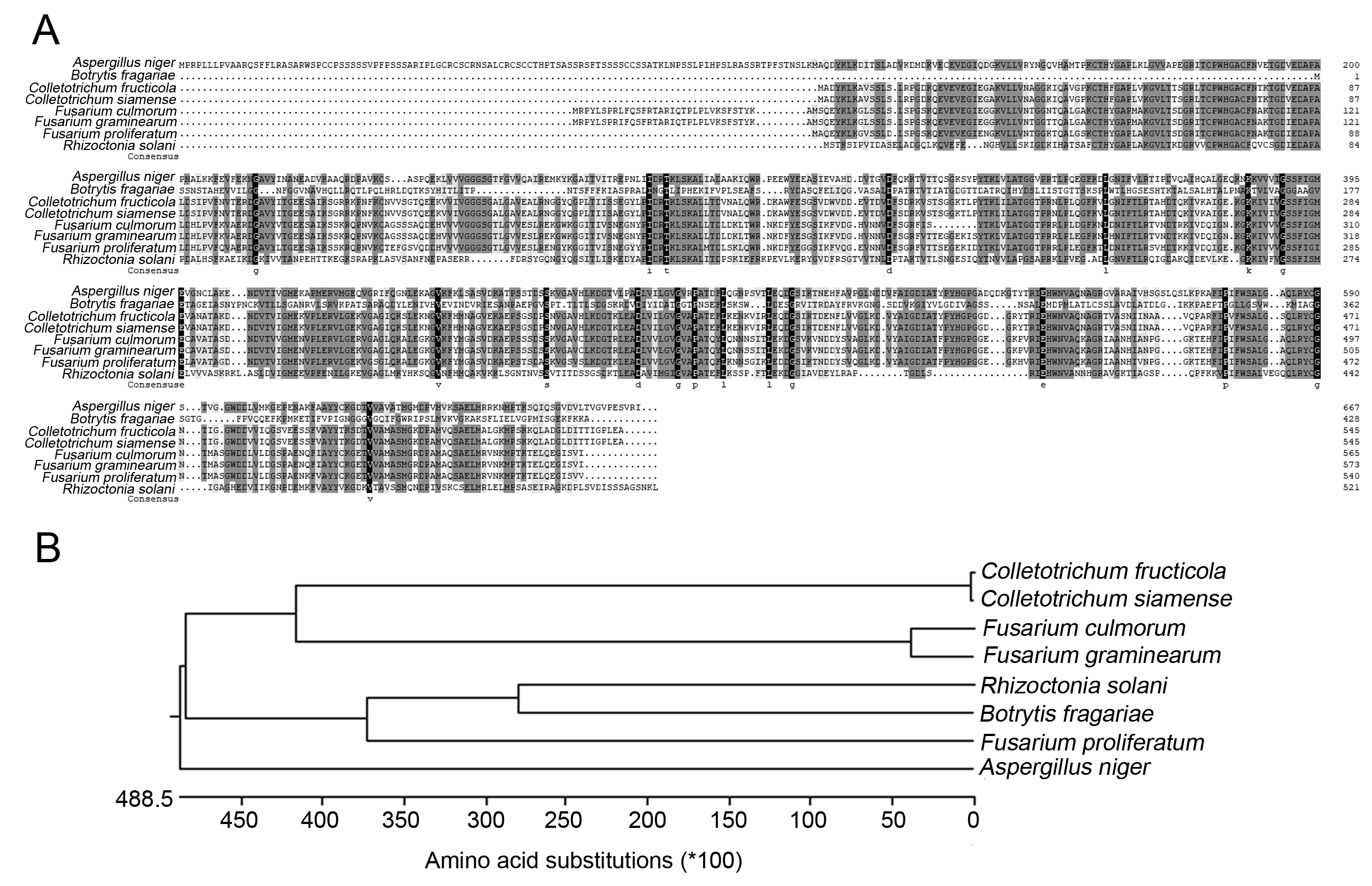

Supplement: Supplementary file 1 [file jof-10-00579-s001.zip › Figure S2.tif]

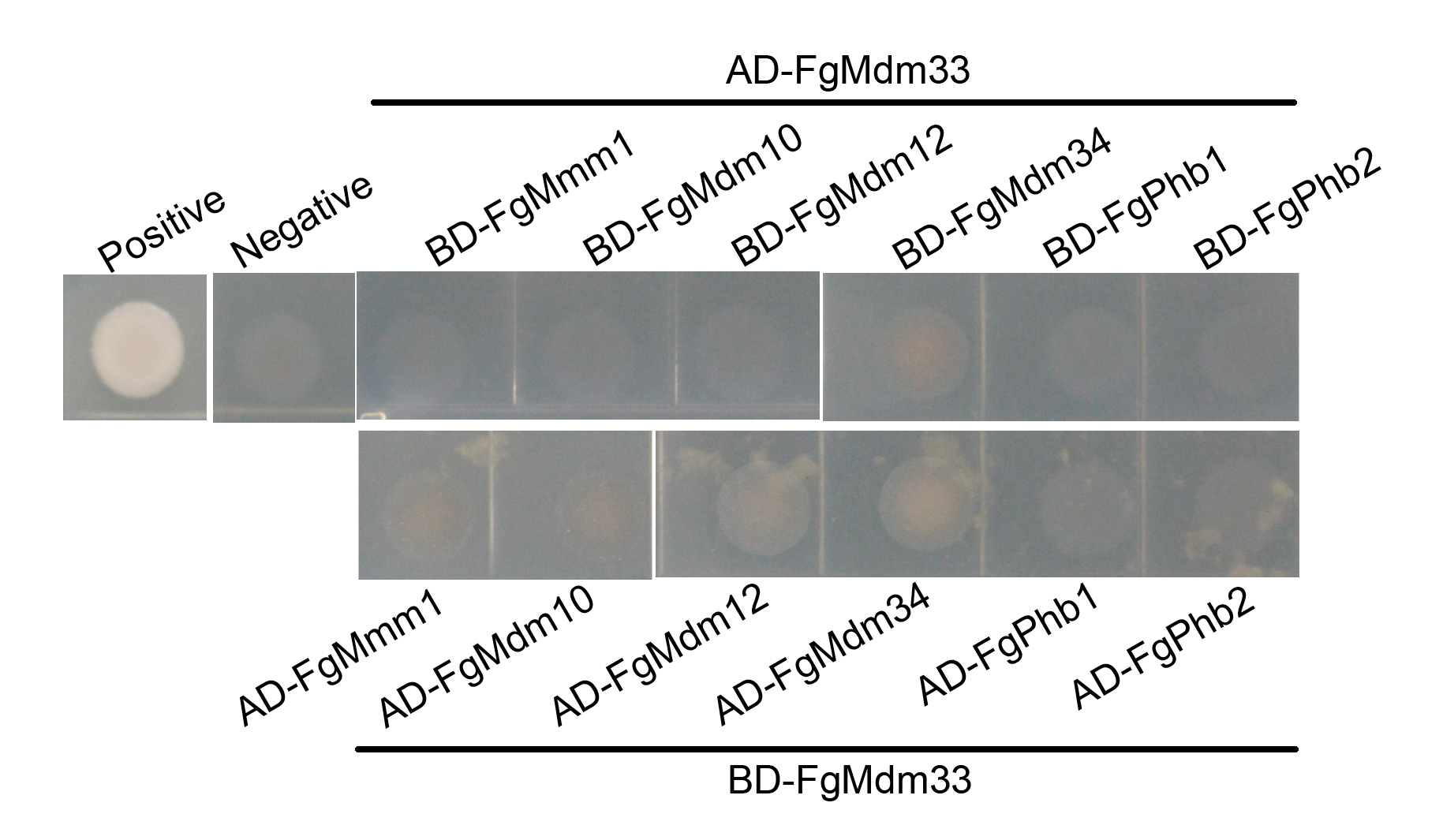

Supplement: Supplementary file 1 [file jof-10-00579-s001.zip › Figure S3.tif]
